# Reliability of Force-Velocity Tests in Cycling and Cranking Exercises in Men and Women

**DOI:** 10.1155/2015/954780

**Published:** 2015-10-11

**Authors:** Hamdi Jaafar, Elvis Attiogbé, Majdi Rouis, Henry Vandewalle, Tarak Driss

**Affiliations:** ^1^Laboratoire CeRSM (EA 2931), Equipe de Physiologie, Biomécanique et Imagerie du Mouvement, UFR STAPS, Université Paris Ouest Nanterre La Défense, 200 avenue de la République, 92000 Nanterre, France; ^2^Laboratoire de Physiologie, UFR de Santé, Médecine et Biologie Humaine, Université Paris XIII, 74 rue Marcel Cachin, 93017 Bobigny, France

## Abstract

The present study examined the reliability of the force-velocity relationship during cycling and arm cranking exercises in active males and females. Twenty male and seventeen female physical education students performed three-session tests with legs and three-session tests with arms on a friction-loaded ergometer on six different sessions in a randomized order. The reliability of maximal power (*P*_max_), maximal pedal rate (*V*_0_), and maximal force (*F*_0_) were studied using the coefficient of variation (CV), the intraclass correlation coefficient (ICC) and the test-retest correlation coefficient (*r*). Reliability indices were better for men (1.74 ≤ CV ≤ 4.36, 0.82 ≤ ICC ≤ 0.97, and 0.81 ≤ *r* ≤ 0.97) compared with women (2.34 ≤ CV ≤ 7.04, 0.44 ≤ ICC ≤ 0.98, and 0.44 ≤ *r* ≤ 0.98) and in cycling exercise (1.74 ≤ CV ≤ 3.85, 0.88 ≤ ICC ≤ 0.98, and 0.90 ≤ *r* ≤ 0.98) compared with arm exercise (2.37 ≤ CV ≤ 7.04, 0.44 ≤ ICC ≤ 0.95, and 0.44 ≤ *r* ≤ 0.95). Furthermore, the reliability indices were high for *P*_max_ and *F*_0_ whatever the expression of the results (raw data or data related to body dimensions). *P*_max_ and *F*_0_ could be used in longitudinal physical fitness investigations. However, further studies are needed to judge *V*_0_ reliability.

## 1. Introduction

Maximal anaerobic power can be measured on friction-loaded cycle ergometers or isokinetic ergometers. Many protocols have been proposed for maximal power measurement: all-out tests against a single load (e.g., the Wingate test) [1, 2], relationship between torque and pedal rate on an isokinetic ergometer [3, 4], relationship between load and peak velocity [5], and force-velocity relationship during a single all-out test against a pure inertial load [6] or inertial + braking load [7–9].

On friction-loaded ergometer, maximal power corresponds to power at peak velocity or is computed during the acceleration phase taking into account the power necessary to increase the flywheel kinetic energy [10]. The relationship between pedal rate (*V*) and braking force (*F*) or torque (*T*) can be described by a linear relationship [3, 5–9, 11]. Linear force-velocity relationships have been described for all-out exercises performed on a cycle ergometer not only with the legs (i.e., cycling exercise) but also with the arms (i.e., cranking exercise). The individual characteristics of the force-velocity or torque-velocity relationship can be defined by two parameters: *V*_0_ (the intercept with the pedal rate axis which has the dimension of a maximal pedal rate) and *F*_0_ or *T*_0_ (the intercepts with the force or torque axis, which have the dimension of a maximal force or a maximal torque). Maximal power (*P*_max⁡_) corresponds to an optimal pedal rate (*V*_opt_) equal to 0.5*V*_0_ and an optimal load or torque equal to 0.5*F*_0_ or 0.5*T*_0_.

Previous studies reported that *P*_max⁡_ [8] or peak power during a Wingate test [12–15] are significantly correlated with the percentage of the fast muscle fibers in the vastus lateralis. Furthermore, a significant positive correlation was observed between *P*_max⁡_ and triceps surae musculotendinous stiffness at relative peak torque corresponding to the optimal cycling rate [16]. On the other hand, the value of *V*_opt_ during sprint cycling was significantly correlated with vastus lateralis myosin heavy chain II composition in a study comparing old and young participants [17]. The proportion of fast twitch fibres expressed in terms of cross-sectional area was highly correlated with *V*_opt_ (*r* = 0.88, *P* < 0.001) [18], and the authors of this latter study suggested that *V*_opt_ would be the most accurate parameter to explore the fibre composition of the knee extensor muscle from cycling tests. The value of *F*_0_ in cycling depends on the strength and the rate of force development of muscle knee extensors [19]. The Wingate optimal braking force can also be determined from the result of a cycling force-velocity test as this braking force is close to 0.5*F*_0_ [5, 20].

Therefore, it could be interesting to determine the parameters of the force-velocity relationships (*V*_0_, *F*_0_, or *T*_0_) in addition to *P*_max⁡_ on a cycle ergometer. Furthermore, the study of the changes in power-velocity relationship during an annual training cycle has been proposed in volleyball players [21], which assumes that the results of the force-velocity tests on cycle ergometers are reliable. The reliability of the cycling all-out tests has mainly been investigated by studying either the test-retest correlation coefficients (*r*_test-retest_) or the intraclass correlation coefficient (ICC) or the standard errors of estimations (SEE) or the coefficients of variation (CV) for the indices of maximal power (Wingate peak power or *P*_max⁡_) with the different protocols [1–4, 6, 9, 22–27]. In contrast, the reliability of the parameters of the force-velocity relationship (slope, *T*_0_, *F*_0_, and *V*_0_) has been investigated in a few studies, only [4, 6, 26]. Moreover, the validity of the statistical tests in these studies on reliability was probably questionable [28].

In a review on the reliability of power in physical performance tests, Hopkins et al. [29] suggested that nonathletic females might be less reliable than nonathletic males, probably because the nonathletic females may be less physically active than the nonathletic males. Similarly, cranking exercises are probably less familiar than cycling exercises and the effect of familiarisation sessions might be more important for force-velocity tests with the arms.

Thus, the aim of the present study was to examine the reliability of *P*_max⁡_, *V*_0_, and *F*_0_ during force-velocity tests. In light of the literature observations, we hypothesized that reliability is lower in women than in men and for cranking force-velocity tests than for cycling tests.

## 2. Materials and Methods

### 2.1. Participants

Twenty healthy males (24.20 ± 2.69 years, 1.80 ± 0.06 m, and 76.48 ± 8.93 kg) and seventeen healthy females (23.53 ± 2.12 years, 1.68 ± 0.06 m, and 61.18 ± 9.58 kg) volunteered to participate in this study. The participants were all active physical education students but none of them were familiarized with sprint cycling or arm cranking before participation in the study. Before any data collection, all participants were fully informed of the possible risk and discomfort associated with the experimental procedures and gave written informed consent. The experimental protocol was approved by the Institutional Review Board of the University and carried out according to the guidelines of the Declaration of Helsinki.

### 2.2. Procedures

The participants performed three session tests with the legs and three session tests with the arms on six different sessions in random order. All the tests were performed within a period of four weeks with at least 48 hours between the sessions. Participants were instructed to avoid any strenuous activity between sessions and to follow their usual diet throughout the experimental period. All tests were performed at the same time of day to minimize the effects of circadian rhythms [30] and with similar standard environmental conditions for all participants (mean temperature and humidity: 22 ± 0.1°C and 35 ± 0.4%, resp.). Body mass and height measures of all subjects were examined before each testing session.

The participants performed a standard warm-up consisting of 5 min cycling (80 W and 50 W for men and women, resp.) before the leg tests or arm cranking (50 W and 20 W for men and women, resp.) for the arm tests, with two short accelerations (3-s) at the end of the third min and the fifth min. After 5 minutes of passive recovery, participants performed the force-velocity test which consisted of repetitive short maximal sprints of 6-s against increasing braking forces. The braking forces administrated at the beginning of the sprints cycling were 2 kg and 1.5 kg for men and women, respectively, while during arm cranking the loads were equal to 1.5 kg and 1 kg for men and women, respectively. Then, the braking force was increased after 5 min of passive recovery (sprints cycling: 2 and 1.5 kg for men and women, resp.; arm cranking: 1.5 and 1 kg for men and women, resp.) until the participant was unable to reach a peak velocity higher than 100 rpm. The same order of braking force application was respected across session tests.

All force-velocity tests were performed on a friction-loaded cycle ergometer with weights (Monark 864, Monark Exercise AB, Vansbro, Sweden) adjustable for both leg and arm exercises [31, 32]. During sprint cycling exercises, participants were seated on the cycle ergometer equipped with toe clips and well-fastened straps to avoid losing the pedals. The same riding position was used throughout the study. Participants were instructed to cycle in seated position to avoid the effect of postural changes [33–35]. During arm cranking exercises, the pedals were replaced with handles and the cycle ergometer was fixed on a metal frame. The participants were standing on their feet in front of the ergometer during the exercises. The center of the pedal axis was approximately 20 cm lower than the level of the shoulder axis. All sprints were performed from the same initial pedal position. Participants were encouraged by the same investigator to reach the maximal velocity rate as quickly as possible. Instantaneous pedal rate in cycling or cranking was monitored throughout a PC computer by means of an encoder placed on the cycle ergometer flywheel. Then, the velocity was averaged over 1-s intervals.

The peak velocity (*V*) was measured for each braking force (*F*) and was used to calculate the linear force-velocity relationship for cycling exercises according to the least squares method:

(1) $$\begin{array}{c} V=a-bF. \end{array}$$

The above relationship was transformed as follows [33]:

(2) $$\begin{array}{c} V=V_{0}\left(1-\frac{F}{F_{0}}\right). \end{array}$$

In this equation, *V*_0_ and *F*_0_ corresponded to the intercepts with the velocity axis and force axis, respectively (*V*_0_ = *a* and *F*_0_ = *a*/*b*). Since a linear relationship between *F* and *V* was assumed, *P*_max⁡_ corresponded to an optimal velocity and an optimal braking force equal to 0.5*V*_0_ and 0.5*F*_0_, respectively. Hence, *P*_max⁡_ was calculated as follows [5, 33]:

(3) $$\begin{array}{c} P_{\max}=0.5V_{0}\times 0.5F_{0}=0.25V_{0}F_{0}. \end{array}$$

The performance variables were expressed in absolute units and according to dimensional scaling. *V*_0_ was expressed in absolute unit (rpm) and relative to body height (rpm · BH^−1^). *F*_0_ was expressed in absolute unit (kg) and relative to body mass raised to the power of 0.67 (kg · BM^−0.67^). *P*_max⁡_ was expressed in absolute unit (W) and relative to body mass (W · BM^−1^).

### 2.3. Relation between the Variabilities of *F*_0_ and *V*_0_

The variability of *F*_0_ and *V*_0_ between the second and first sessions (Δ*F*_0  2-1_ and Δ*V*_0  2-1_) and between the third and second sessions (Δ*F*_0  3-2_ and Δ*V*_0  3-2_) was calculated according to the following formulas:

(4) $$\begin{array}{c} \Delta F_{0\;2\text{-}1}=100\frac{F_{02}}{F_{01}},\\ \Delta F_{0\;3\text{-}2}=100\frac{F_{03}}{F_{02}},\\ \Delta V_{0\;2\text{-}1}=100\frac{V_{02}}{V_{01}},\\ \Delta V_{0\;3\text{-}2}=100\frac{V_{03}}{V_{02}}. \end{array}$$

### 2.4. Statistical Analyses

Statistical procedures were carried out using Statistica 7.1 Software (StatSoft, France). Data of *V*_0_, *F*_0_, and *P*_max⁡_ are presented as mean and standard deviation (mean ± SD). Before statistical analysis, each performance variable was tested for normality with the Shapiro-Wilk test. With the assumption of normality confirmed, systematic change in performance from trials 1 to 3 was examined using one-way ANOVA with repeated measures and a Tukey's post hoc test. All significance thresholds were set at *P* < 0.05.

Absolute reliability, which concerns the consistency of individual's scores [36], was determined using the standard error of measurement SEM and the coefficient of variation (CV) using the following formulas [37]:

(5) $$\begin{array}{c} \text{SEM}=\frac{{\text{SD}}_{\text{diff}}}{\sqrt{2}},\\ \text{CV}\left(\%\right)=\frac{\text{SEM}}{\text{Mean}}\times 100, \end{array}$$

where SD_diff_ was the standard deviation of the differences between consecutive session tests (i.e., sessions 1 and 2 and sessions 2 and 3).

Relative reliability, which concerns the consistency of individual's position in the group relative to others [36], was assessed using the intraclass correlation coefficient of two-way random effects model with single measure for each pair of consecutive session tests (i.e., sessions 1 and 2 and sessions 2 and 3) as follows:

(6) $$\begin{array}{c} \text{ICC}\left(2,1\right)=\frac{{\text{MS}}_{P}-{\text{MS}}_{E}}{{\text{MS}}_{P}+{\text{MS}}_{E}+2\left({\text{MS}}_{T}-{\text{MS}}_{E}\right)/n}. \end{array}$$

In this formula MS_*P*_ represents the participant mean square, MS_*E*_ represents the error mean square, *k* is the number of trials, MS_*T*_ represents the trials mean square, and *n* is the number of participants. The ICC is considered as high for values above 0.90, moderate for values between 0.80 and 0.90, and low for values below 0.80 [38].

In addition, the test-retest correlation coefficient (*r*_test-retest_) was calculated for each pair of consecutive session tests in order to compare the results of the present study to the data in the literature [29]. The Bland-Altman plots were used to check for heteroscedasticity [28].

## 3. Results

### 3.1. Variations in Body Mass (BM)

For the arm tests, the differences in BM between the sessions were equal to −0.08 ± 0.754 (Δ*S*2 − *S*1), 0.305 ± 0.669 (Δ*S*3 − *S*2), and 0.225 ± 0.916 kg (Δ*S*3 − *S*1) in men and 0.129 ± 0.512 (Δ*S*2 − *S*1), 0.006 ± 0.553 (Δ*S*3 − *S*2), and 0.124 ± 0.529 kg (Δ*S*3 − *S*1) in women.

For the leg tests, the differences in BM between the sessions were equal to 0.090 ± 0.704 (Δ*S*2 − *S*1), 0.255 ± 0.737 (Δ*S*3 − *S*2), and 0.345 ± 0.944 kg (Δ*S*3 − *S*1) in men and 0.288 ± 0.499 (Δ*S*2 − *S*1), −0.206 ± 0.536 (Δ*S*3 − *S*2), and 0.08 ± 0.591 kg (Δ*S*3 − *S*1) in women.

### 3.2. *V*_0_, *F*_0_, and *P*_max⁡_ in the Three Sessions

**Figure 1:**
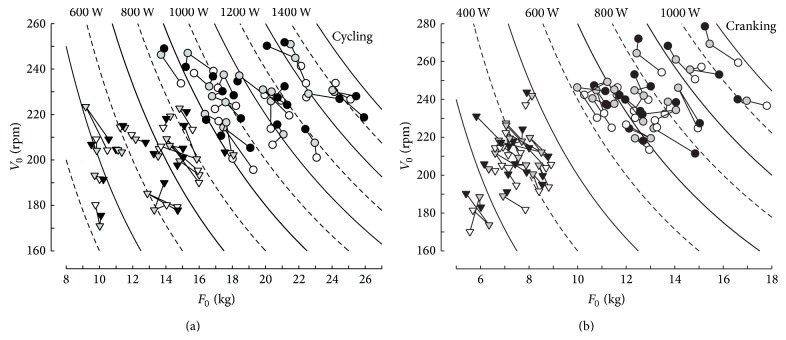
Individual values of *V*_0_ and *F*_0_ corresponding to the force-velocity relationships in cycling (a) and cranking (b) at the first (empty symbols), second (grey symbols), and third (black symbols) trials. The three values of each participant are linked by broken lines. Circles and triangles correspond to men and women, respectively.

**Figure 2:**
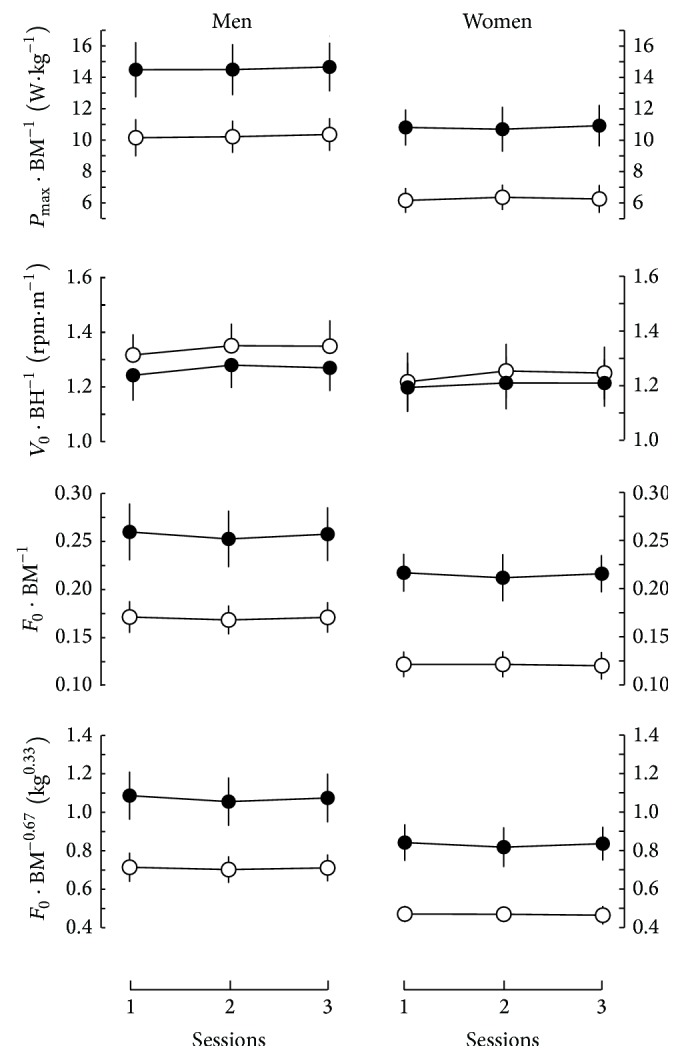
Results of the force-velocity tests (means ± SD) in the three sessions related to body dimensions (*F*_0_ related to BM and BM^−0.67^, *V*_0_ related to BH, and *P*_max⁡_ related to BM). Black points, exercises performed with the legs; empty circle, exercises performed with the arms.

The individual values of *F*_0_ and *V*_0_ measured in the three sessions are presented in Figure 1. The branches of hyperbolae (i.e., continuous and dashed curves) in Figure 1 correspond to the participants with different combinations of *F*_0_ and *V*_0_ but the same value of *P*_max⁡_. The means ± SD and ranges of *P*_max⁡_, *F*_0_, *V*_0_, *P*_max⁡_ · BM^−1^, *F*_0_ · BM^−1^, *F*_0_ · BM^−0.67^, and *V*_0_ · BH^−1^ measured in the different sessions are presented in Tables 1 and 2 and Figures 1 and 2. In Table 1 and Figure 1, BM corresponded to the body mass measured during each session whereas BM was equal to the average of the three measures of BM in Figure 2.

All the differences between men and women were highly significant (*P* < 0.001) even when the data were related to body mass (*P*_max⁡_ · BM^−1^, *F*_0_ · BM^−1^, and *F*_0_ · BM^−0.67^). The significance level of the difference in *V*_0_ · BH^−1^ between men and women was equal to *P* < 0.05, only.

### 3.3. Reliability

The one-way ANOVA with repeated measure showed a significant main effect of trial on *V*_0_ in men (*F*_(2,38)_ = 11.48, *P* < 0.001 and *F*_(2,38)_ = 6.93, *P* < 0.01, for cycling and cranking, resp.) and women (*F*_(2,32)_ = 4.55, *P* < 0.05 and *F*_(2,32)_ = 6.10, *P* < 0.01, for cycling and cranking, resp.). Tukey's post hoc tests revealed that *V*_0_ at session 1 was significantly lower by comparison to sessions 2 and 3. In contrast, there was no significant main effect of sessions on *F*_0_ and *P*_max⁡_ for arms and legs in men and women (*P* > 0.05).

The CV (%) of *V*_0_, *F*_0_, and *P*_max⁡_ are presented in Tables 3 and 4. The highest CV values were obtained for *F*_0_ by comparison with *V*_0_ and *P*_max⁡_. The greatest CV values were observed for cranking exercises in female participants.

The values of *r*_test-retest_ are presented in Tables 3 and 4. The values of *r*_test-retest_ increased for the correlations between sessions 2 and 3 when compared with the correlations between sessions 1 and 2. Except *F*_0_ with the arms in women, the lowest *r*_test-retest_ were observed for *V*_0_.

For the correlations between the results of the first and second sessions, the values of *r*_test-retest_ for *F*_0_ were significantly different between cycling and cranking but in the female group, only (*P* = 0.030 for *F*_0_; *P* = 0.036 for *F*_0_ related to BM^−0.67^). Similarly, the values of *r*_test-retest_ between the first and second sessions were significantly different between male and female groups for *F*_0_ and *P*_max⁡_ (*P* = 0.007 for *F*_0_, *P* = 0.005 for *F*_0_ related to BM^−0.67^, and *P* = 0.047 for *P*_max⁡_ in watts). For the correlations between the results of the second and third sessions, the values of *r*_test-retest_ for *F*_0_ and *P*_max⁡_ were significantly different between cycling and cranking but in the female group, only (*P* = 0.01 for *F*_0_; *P* = 0.006 for *F*_0_ related to BM^−0.67^ and *P* = 0.023 for *P*_max⁡_ in watts). All the other comparisons of *r*_test-retest_ between men and women or cycling and cranking were not significantly different.

The ICC of each performance variable across sessions 1 and 2 and sessions 2 and 3 in male and female participants are presented in Tables 3 and 4. The values of ICC improved for sessions 2 and 3 by comparison with sessions 1 and 2. Excepting *F*_0_ with the arms in female participants, the lowest ICC values were observed for *V*_0_.

### 3.4. Relation between the Variabilities of *F*_0_ and *V*_0_

**Figure 3:**
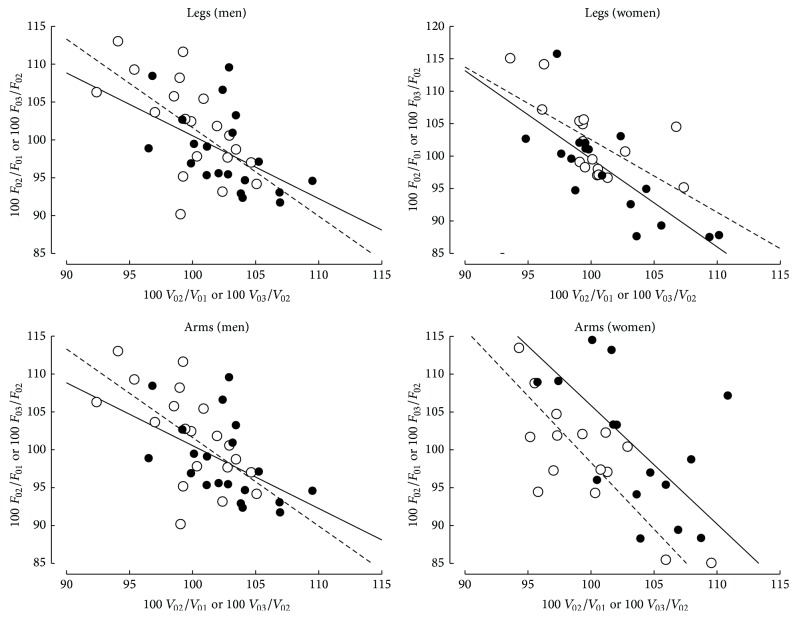
Relationships between intersession differences in *F*_0_ (ordinates) and intersession differences in *V*_0_ (abscissae) for the leg and arm force-velocity tests in men and women. Continuous lines and black points: differences between the first and second sessions. Dashed line and empty circles: differences between the second and third sessions.

The variability of *F*_0_ (Δ*F*_0  2-1_ or Δ*F*_0  3-2_) was significantly correlated with the variability of *V*_0_ (Δ*V*_0  2-1_ or Δ*V*_0  3-2_) as shown in Figure 3:

  in women:
  (7) ΔF0arms2-1=263−1.57 ΔV0arms2-1,r=0.695; P=0.002,ΔF0arms3-2=274−1.76 ΔV0arms3-2,r=0.742; P<0.001,ΔF0legs2-1=235−1.36 ΔV0legs2-1,r=0.773; P<0.001,ΔF0legs3-2=215−1.12 ΔV0legs3-2,r=0.644; P=0.005,
  in men:
  (8) ΔF0arms2-1=184−0.83 ΔV0arms2-1,r=0.503; P=0.024,ΔF0arms3-2=219−1.17 ΔV0arms3-2,r=0.624; P=0.003,ΔF0legs2-1=184−0.83 ΔV0legs2-1,r=0.503; P=0.024,ΔF0legs3-2=219−1.17 ΔV0legs3-2,r=0.624; P=0.003.

## 4. Discussion

In the present investigation, we studied the reliability of *P*_max⁡_, *V*_0_, and *F*_0_ during cycling and arm cranking exercises in active men and women. In order to study the reliability of these parameters, force-velocity tests on cycle ergometer were separately repeated three times in different sessions for each exercise. It was assumed that reliability was lower (1) in women than in men and (2) for cranking force-velocity tests than for cycling tests. The results of the present study were in agreement with this hypothesis: the reliability indices were better for the men and the leg indices when compared with the women and arm indices (Tables 3 and 4). Whatever the force-velocity parameter (*V*_0_, *F*_0_, and *P*_max⁡_), familiarisation sessions might be more important for women and arm tests as indicated by the lower values of CV in men and leg tests when the results of the first and second sessions were compared (Table 3).

The reliability of *P*_max⁡_ was similar to the reliability of the different indices of maximal power in previous studies. For example, the reliability of the results of the Wingate is good for the peak power (*r*_test-retest_ > 0.90) and the mean power (*r*_test-retest_ between 0.91 and 0.93) [1, 2, 22], in contrast with the reliability of the fatigue index (*r*_test-retest_ = 0.43). Similarly, the reliability of the power indices measured with the different force-velocity protocols was high when measured with isokinetic cycle ergometers [3, 4, 9], friction-loaded ergometers [23, 24, 26], or the inertial load method [6, 25]. In a study by Winter et al. [23], the maximal power computed during the acceleration phase (*PP*_corr_) estimated according to Lakomy [10] was 10% higher than *P*_max⁡_ but the reliability of *PP*_corr_ was lower (*r*_test-retest_: 0.530 for *PP*_corr_ versus 0.972 for *P*_max⁡_ in men, and 0.922 for *PP*_corr_ versus 0.952 for *P*_max⁡_ in women). In the same study of Winter et al. [23], the CV values of *PP*_corr_ were higher in men (6.9% for *PP*_corr_ versus 2.7% for *P*_max⁡_) but not in women (3.7% for *PP*_corr_ versus 4.2% for *P*_max⁡_). Furthemore, according to Winter et al. [23], these results of optimization procedures (i.e., the method of Vandewalle et al. [5]) add further support and have securer fundations than those enjoyed by correction procedures [10]. For arm exercises, Smith et al. [39] reported CV values of 4.5% for *PP*_corr_ and 2.8% for *P*_max⁡_. It is likely that the lower reliability of *PP*_corr_ is explained by oscillations of *P*_corr_ (product of *V* and *F*_corr_ that takes into account not only the braking force but also the force necessary for the flywheel acceleration). On isokinetic cycle ergometers, the coefficients of variation of the slope and intercept of the regression between torque and pedal rate were 13.7 and 10.5%, respectively [4].

The values of CV of *V*_0_, *F*_0_, and *P*_max⁡_ in the present study were similar to the values of CV for the different parameters measured with the inertial method (4 trials on the same day): 3.3% for *PP*_corr_, 2.7% for *V*_0_, and 4.4% for *T*_0_ [6]. For friction-loaded ergometers, the reliability of the force-velocity parameters in cycling has been tested in male physical education students [26]. For *F*_0_ and *P*_max⁡_, SEE was lower than 5% and *r*_test-retest_ or ICC were higher than 0.90 as in the present study for the cycling force-velocity test in the male participants. However, the comparison and the validity of the reliability indices must take into account the characteristics of the data [28, 37]. The data are said to be homoscedastic when the random error does not depend on the size of the measured value. Homoscedastic errors are generally expressed in the same units as those of their measurements and they can be analysed with conventional parametric analyses. SEM is valid when the data are homoscedastic. The data are said to be heteroscedastic when the random error increases as the measured values increase. Heteroscedastic data should be measured on a ratio scale (e.g., percentage) and be investigated with an analysis based on nonparametric analyses (i.e., rank tests). CV is valid even when the data are heteroscedastic. The heterogeneity of values between participants influences the results of the reliability tests.

(1) The coefficient of correlation of test-retest (*r*_test-retest_) is sensitive to the heterogeneity of data between participants.
(2) The effect of heteroscedascity on the observed “errors” in a test-retest is low when the data range is narrow.

**Figure 4:**
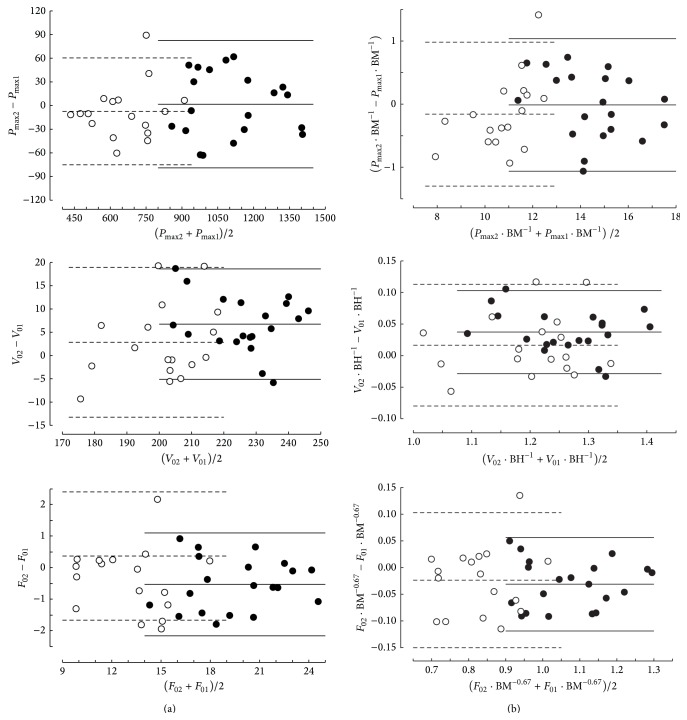
Bland and Altman plots of the results of differences in parameters *P*_max⁡_, *V*_0_, and *F*_0_ ((a) raw data; (b) data related to body dimensions) between sessions 1 and 2, in men (black points and continuous lines) and women (empty circles and dashed lines).

**Figure 5:**
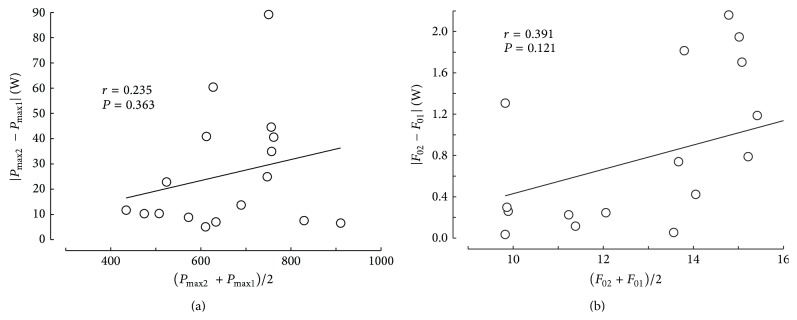
Plot of the absolute differences between the results of sessions 1 and 2 (ordinates) and the individual means (abscissae) for *P*_max⁡_ (a) and *F*_0_ (b) in women.

The spread of the data between participants is different for *V*_0_, *F*_0_, and *P*_max⁡_ expressed in percentage of the group averages even when they are related to body dimension (Table 5). Heteroscedasticity was expected for *V*_0_, *F*_0_, and *P*_max⁡_ raw data. However, this expectation was not confirmed with Bland-Altman plots of these data, especially in men (Figure 4). The data ranges of parameters *V*_0_, *F*_0_, and *P*_max⁡_ were lower than 62% in men (Table 5), which could partly explain that heteroscedascity was not suggested by the Bland-Altman plots of *V*_0_, *F*_0_, and *P*_max⁡_ raw data (Figure 4). In women, the data ranges were larger than in men when the ranges were expressed as percentages of the means (Table 5) but the correlations of the absolute values of the differences versus the means of the results in the first and second sessions (Figure 5) were not significant. All other things being equal, the differences between sessions are probably lower in well-motivated individuals and experts in cycling and the average of their performances in sessions 1 and 2 should be higher (and inversely for the nonexperts and not motivated individuals). Therefore, the effects of motivation and expertise can alter the results of the Bland-Altman plot in this kind of physical tests.

As in the study by Attiogbé et al. [26], the values of *r*_test-retest_ and ICC were lower for *V*_0_ than for *F*_0_ and *P*_max⁡_, which can be partly explained by the smaller variance of this parameter. Indeed, the range of *V*_0_ is smaller (Table 5) than the range of *F*_0_ and *P*_max⁡_. The small variance of *V*_0_ in the present study is probably an expression of the small variance of *V*_0_ when compared with the variances of *F*_0_ and *P*_max⁡_ in a general athletic population [35]. The small range of *V*_0_ also probably explains that the values of CV in men and women were lower for *V*_0_ than for *F*_0_ and *P*_max⁡_ in the cycling as well as the cranking force-velocity tests. Excepting the study by Buśko [21], there is no data about the changes in *V*_0_ during an annual training cycle and, therefore, it is difficult to know whether its reliability is good enough for the estimation of the training effect on this parameter.

The ranges of *F*_0_ and *P*_max⁡_ were similar but the values of *r*_test-retest_ or ICC were higher for *P*_max⁡_ than for *F*_0_ (and *V*_0_). It is likely that the variations in *V*_0_ and *F*_0_ between sessions are not totally independent (Figure 3). Indeed, the values of *V*_0_ and *F*_0_ are extrapolated from the relationship between braking force and peak velocity. An underestimation of the peak velocity corresponding to the highest braking force induces a rotation of the F-V regression line (i.e., a more negative slope) and, consequently, an overestimation of *V*_0_ in addition to an underestimation of *F*_0_. Inversely, an underestimation of the peak velocity corresponding to the lowest braking force induces a less negative slope of the *F*-*V* regression line and, consequently, and overestimation of *F*_0_ in addition to an underestimation of *V*_0_. The value of *P*_max⁡_ depends on *F*_0_ and *V*_0_ and the effect of an underestimation of *V*_0_ on *P*_max⁡_ should be compensated by the effect of an overestimation of *F*_0_, and* vice versa*. This could partly explain why the values of *r*_test-retest_, ICC, or CV were better for *P*_max⁡_ than for *F*_0_.

The values of *V*_0_, *F*_0_, and *P*_max⁡_ were lower in women than in men. The differences in BH and BM were not the only explanations of the lower values of *V*_0_, *F*_0_, and *P*_max⁡_ in women. Indeed, these differences were still significant when force-velocity parameters were related to BH or BM (*V*_0_ · BH^−1^, *F*_0_ · BM^−0.67^, and *P*_max⁡_ · BM^−1^). This gender effect could partly be explained by a difference in muscle fiber composition as, for example, the higher percentage of the cross-sectional area that corresponds to the slow fibers in women [40–42]. The lower values of *F*_0_ · BM^−0.67^, *F*_0_ · BM^−1^, and *P*_max⁡_ · BM^−1^ might partly be explained by a lower percentage of lean body mass in women. The lower values of *r*_test-retest_ in women cannot be explained by a lower range of the individual data (Table 5). The lower reliability in women might partly be explained by the effect of menstrual cycle, but it is possible that this effect is less important in trained women because training might reduce the cyclical hormonal fluctuations [29].

The variability of *F*_0_ and *P*_max⁡_ depends on the variability of BM when these data are related to body mass (*F*_0_ · BM^−1^, *F*_0_ · BM^−0.67^, and *P*_max⁡_ · BM^−1^). In spite of the instructions about diet, hydration, and training, the standard deviations of the differences in BM between the sessions were not negligible (<1.25% of BM).

## 5. Methodological Considerations

To the best of our knowledge, this is the first study examining the reliability of force-velocity tests on cycle ergometer during sprint cycling and arm cranking exercises in active men and women. One of the limitations inherent to the experimental protocol in the present study is that the crank length was the same for all participants. The usual crank length is probably higher than the optimal length in small participants, which could partially explain the lower reliability in women. Therefore, familiarization sessions are required in small participants.

## 6. Conclusion

The present study showed high reliability of *P*_max⁡_ and *F*_0_, allowing the use of these parameters in longitudinal evaluations. Furthermore, the reliability of *P*_max⁡_ was better than that of *F*_0_ whatever the expression of the results (expressed in absolute unit or data related to body dimension). The reliability indices were also better in men and cycling force-velocity tests than in women and cranking force-velocity tests. Further studies are needed to judge the reliability of *V*_0_.

## Figures and Tables

**Table 1:** Parameters *P*_max⁡_, *F*_0_, and *V*_0_ (means, SD, and range) computed from the force-velocity tests performed with legs or arms by men in sessions 1, 2, and 3.

			Session 1	Session 2	Session 3
Legs	*V*_0_	rpm	223 ± 14 (196–241)	230 ± 12 (208–251)	228 ± 13 (205–242)
		rpm·BH^−1^	1.24 ± 0.09 (1.08–1.38)	1.28 ± 0.08 (1.11–1.43)	1.27 ± 0.08 (1.14–1.45)
	*F*_0_	kg	19.8 ± 2.9 (14.9–25.1)	19.3 ± 3.0 (13.7–24.1)	19.7 ± 3.3 (13.9–25.9)
		kg·BM^−1^	0.26 ± 0.03 (0.21–0.32)	0.25 ± 0.03 (0.21–0.31)	0.26 ± 0.03 (0.22–0.31)
		kg·BM^−0.67^	1.09 ± 0.12 (0.89–1.30)	1.06 ± 0.13 (0.88–1.29)	1.07 ± 0.13 (0.88–1.33)
	*P*_max⁡_	W	1105 ± 174 (871–1423)	1107 ± 173 (844–1387)	1122 ± 182 (865–1451)
		W·BM^−1^	14.5 ± 1.8 (11.4–17.7)	14.5 ± 1.6 (11.4–17.6)	14.6 ± 1.5 (11.8–17.8)

Arms	*V*_0_	rpm	237 ± 12 (213–259)	243 ± 14 (219–269)	242 ± 17 (211–279)
		rpm·BH^−1^	1.32 ± 0.08 (1.21–1.46)	1.35 ± 0.08 (1.24–1.48)	1.35 ± 0.09 (1.20–1.52)
	*F*_0_	Kg	13.1 ± 1.9 (10.2–17.8)	12.9 ± 1.7 (10.0–17.0)	13.1 ± 1.7 (10.7–16.6)
		kg·BM^−1^	0.17 ± 0.02 (0.14–0.21)	0.17 ± 0.02 (0.13–0.20)	0.17 ± 0.02 (0.14–0.21)
		kg·BM^−0.67^	0.71 ± 0.08 (0.57–0.86)	0.70 ± 0.07 (0.55–0.81)	0.71 ± 0.07 (0.60–0.86)
	*P*_max⁡_	W	777 ± 136 (620–1077)	781 ± 122 (615–1039)	792 ± 123 (660–1061)
		W·BM^−1^	10.1 ± 1.2 (8.2–12.1)	10.2 ± 1.1 (8.2–12.3)	10.3 ± 1.1 (8.4–12.0)

**Table 2:** Parameters *P*_max⁡_, *F*_0_, and *V*_0_ (means, SD, and range) computed from the force-velocity tests performed with legs or arms by women in sessions 1, 2, and 3.

			Session 1	Session 2	Session 3
Legs	*V*_0_	rpm	200 ± 12 (179–215)	203 ± 15 (171–223)	203 ± 13 (176–221)
		rpm·BH^−1^	1.19 ± 0.09 (1.00–1.35)	1.21 ± 0.10 (1.04–1.36)	1.21 ± 0.09 (1.00–1.32)
	*F*_0_	kg	13.3 ± 2.6 (9.8–17.9)	12.9 ± 2.4 (9.2–18.1)	13.2 ± 2.3 (9.5–17.5)
		kg·BM^−1^	0.22 ± 0.02 (0.19–0.25)	0.21 ± 0.02 (0.16–0.26)	0.21 ± 0.02 (0.19–0.25)
		kg·BM^−0.67^	0.84 ± 0.09 (0.69–1.01)	0.82 ± 0.10 (0.69–1.01)	0.83 ± 0.09 (0.69–0.99)
	*P*_max⁡_	W	662 ± 130 (430–907)	655 ± 136 (428–914)	668 ± 131 (443–893)
		W·BM^−1^	10.8 ± 1.1 (8.3–12.4)	10.7 ± 1.4 (7.5–12.9)	10.9 ± 1.3 (8.4–13.3)

Arms	*V*_0_	rpm	203 ± 17 (170–237)	210 ± 16 (174–242)	209 ± 16 (183–244)
		rpm·BH^−1^	1.21 ± 0.11 (1.03–1.37)	1.25 ± 0.10 (1.07–1.41)	1.25 ± 0.10 (1.41–1.10)
	*F*_0_	kg	7.4 ± 1.0 (5.6–9.0)	7.3 ± 0.8 (6.0–8.5)	7.3 ± 1.0 (5.4–8.7)
		kg·BM^−1^	0.12 ± 0.01 (0.10–0.14)	0.12 ± 0.01 (0.09–0.15)	0.12 ± 0.01 (0.09–0.14)
		kg·BM^−0.67^	0.47 ± 0.04 (0.39–0.54)	0.47 ± 0.04 (0.38–0.55)	0.46 ± 0.05 (0.38–0.54)
	*P*_max⁡_	W	375 ± 61 (237–466)	386 ± 59 (276–491)	380 ± 63 (257–482)
		W·BM^−1^	6.2 ± 0.8 (4.6–7.7)	6.4 ± 0.8 (5.1–7.6)	6.3 ± 0.9 (4.9–7.7)

**Table 3:** Differences between sessions 1 and 2; coefficients of variation (CV), intraclass correlation coefficients (ICC), and test-retest correlation coefficients (*r*_test-retest_) for *V*_0_, *F*_0_, and *P*_max⁡_ for the leg or arm force-velocity tests in men and women.

			Men		Women	
			Legs	Arms	Legs	Arms
SEM	*V*_0_	rpm	4.28	5.30	5.80	6.67
		rpm·BH^−1^	0.02	0.03	0.03	0.04
	*F*_0_	kg	0.59	0.48	0.73	0.58
		kg·BM^−0.67^	0.03	0.03	0.05	0.04
	*P*_max⁡_	W	29.10	24.9	24.5	21.7
		W·BM^−1^	0.38	0.32	0.41	0.35

CV (%)	*V*_0_	rpm	1.89	2.21	2.88	3.23
		rpm·BH^−1^	1.89	2.25	2.90	3.23
	*F*_0_	kg	3.01	3.69	5.60	7.84
		kg·BM^−0.67^	2.95	3.75	5.50	7.52
	*P*_max⁡_	W	2.63	3.19	3.71	5.69
		W·BM^−1^	2.61	3.18	3.83	5.60

ICC	*V*_0_	rpm	0.79	0.75	0.80	0.78
		rpm·BH^−1^	0.93	0.78	0.85	0.80
	*F*_0_	kg	0.95	0.93	0.91	0.60
		kg·BM^−0.67^	0.91	0.86	0.77	0.25
	*P*_max⁡_	W	0.97	0.96	0.97	0.86
		W·BM^−1^	0.95	0.93	0.90	0.79

*r*_test-retest_	*V*_0_	rpm	0.89	0.84	0.82	0.84
		rpm·BH^−1^	0.93	0.85	0.86	0.85
	*F*_0_	kg	0.96	0.94	0.91	0.60
		kg·BM^−0.67^	0.94	0.87	0.79	0.24
	*P*_max⁡_	W	0.97	0.97	0.97	0.87
		W·BM^−1^	0.95	0.93	0.92	0.80

**Table 4:** Differences between sessions 2 and 3; coefficients of variation (CV), intraclass correlation coefficients (ICC), and test-retest correlation coefficients (*r*_test-retest_) for *V*_0_, *F*_0_, and *P*_max⁡_ for the leg or arm force-velocity tests in men and women.

			Men		Women	
			Legs	Arms	Legs	Arms
SEM	*V*_0_	rpm	3.97	5.74	4.76	6.01
		rpm·BH^−1^	0.02	0.03	0.03	0.04
	*F*_0_	kg	0.65	0.56	0.50	0.51
		kg·BM^−0.67^	0.01	0.03	0.03	0.03
	*P*_max⁡_	W	29.8	26.3	19.1	20.6
		W·BM^−1^	0.38	0.32	0.27	0.33

CV	*V*_0_	rpm	1.74	2.37	2.35	2.87
		rpm·BH^−1^	1.74	2.37	2.34	2.91
	*F*_0_	kg	3.34	4.36	3.85	7.01
		kg·BM^−0.67^	3.26	4.21	3.56	7.04
	*P*_max⁡_	W	2.67	3.35	2.88	5.37
		W·BM^−1^	2.63	3.16	2.50	5.17

ICC	*V*_0_	rpm	0.90	0.87	0.88	0.86
		rpm·BH^−1^	0.93	0.87	0.91	0.87
	*F*_0_	kg	0.95	0.89	0.95	0.69
		kg·BM^−0.67^	0.92	0.82	0.90	0.44
	*P*_max⁡_	W	0.97	0.95	0.98	0.89
		W·BM^−1^	0.94	0.92	0.95	0.85

*r*_test-retest_	*V*_0_	rpm	0.90	0.88	0.89	0.86
		rpm·BH^−1^	0.93	0.88	0.91	0.86
	*F*_0_	kg	0.96	0.89	0.96	0.70
		kg·BM^−0.67^	0.92	0.81	0.92	0.44
	*P*_max⁡_	W	0.97	0.95	0.98	0.89
		W·BM^−1^	0.94	0.92	0.97	0.85

**Table 5:** Ranges of parameters *V*_0_, *F*_0_, and *P*_max⁡_ expressed in percentage of the means of the male or female groups.

		Men			Women		
		Session 1	Session 2	Session 3	Session 1	Session 2	Session 3
Legs	*V*_0_	20.5	18.9	20.4	17.9	25.9	22.6
	*V*_0_·BH^−1^	24.7	25.2	24.1	28.9	26.4	27.0
	*F*_0_	51.5	54.0	61.0	61.0	68.9	61.0
	*F*_0_·BM^−0.67^	38.1	38.6	41.8	37.7	43.6	35.9
	*P*_max⁡_	50.0	49.0	52.3	70.5	74.1	67.3
	*P*_max⁡_·BM^−1^	43.5	42.4	40.9	37.7	51.0	45.3

Arms	*V*_0_	19.4	20.6	27.7	32.9	32.5	29.1
	*V*_0_·BH^−1^	19.5	18.0	24.5	27.9	27.6	24.6
	*F*_0_	57.6	54.3	45.3	46.4	34.7	45.8
	*F*_0_·BM^−0.67^	40.9	37.0	37.1	30.6	36.7	33.8
	*P*_max⁡_	58.8	54.4	50.7	61.1	55.7	59.1
	*P*_max⁡_·BM^−1^	37.7	40.1	35.9	51.0	39.9	45.1
